# Autophagic flux in cancer cells at the invasive front in the tumor-stroma border

**DOI:** 10.18632/aging.203406

**Published:** 2021-08-17

**Authors:** Fei Yan, Xuexi Zhang, Rongying Tan, Mingchen Li, Zengtuan Xiao, Hao Wang, Zhenfa Zhang, Zhenyi Ma, Zhe Liu

**Affiliations:** 1 Department of Immunology, Biochemistry and Molecular Biology, Collaborative Innovation Center of Tianjin for Medical Epigenetics, Key Laboratory of Immune Microenvironment and Disease of the Ministry of Education, State Key Laboratory of Experimental Hematology, Tianjin Medical University, Tianjin, China; 2 Department of Lung Cancer Center, Tianjin Medical University Cancer Institute and Hospital, Tianjin, China

**Keywords:** autophagy, invasive front, invasion, lung cancer, tumor-stroma border

## Abstract

Cancer cells at the invasive front directly interact with stromal tissue that provides a microenvironment with mechanical, nutrient, and oxygen supply characteristics distinct from those of intratumoral tissues. It has long been known that cancer cells at the invasive front and cancer cells inside the tumor body exhibit highly differentiated functions and behaviors. However, it is unknown whether cancer cells at different locations exhibit a variety of autophagic flux, an important catabolic process to maintain cellular homeostasis in response to environmental changes. Here, using transmission electron microscopy (TEM), we found that invading cancer cells at the invasive front, which show mesenchymal transcriptomic traits, exhibit higher autophagic flux than cancer cells inside the tumor body in human primary non-small cell lung cancer (NSCLC) tissues. This autophagic feature was further confirmed by a live cell autophagic flux monitoring system combined with a 3D organotypic invasion coculture system. Additionally, the increased autophagic flux endows cancer cells with invasive behavior and positively correlates with the advanced tumor stages and the reduced survival period of lung cancer patients. These findings expand the understanding of autophagic dynamics during cancer invasion.

## INTRODUCTION

Metastasis is the leading cause of cancer-related deaths. Metastasis begins with local invasion, whereby cancer cells at the primary site invade the surrounding tissues and migrate towards the vascular systems. Solid tumors are highly heterogeneous and can often be anatomically classified into areas of the leading edge and tumor body [1–3]. Cancer cells at different locations are exposed to microenvironments with distinct mechanical, nutrient, oxygen supply, and pH characteristics [4], which affect their transcriptomics, metabolism, and signaling cascades and consequently resulting in dysregulated cellular behaviors [5–7]. Reports have shown that cancer cells at the leading edge are more invasive than cancer cells inside the tumor body [8–10].

Macroautophagy/autophagy (hereafter referred to as autophagy), a multistep lysosomal degradation pathway that supports nutrient recycling and metabolic adaptation, has been considered as a process that regulates cancer survival during stresses [11]. Although autophagy induction may inhibit tumors initiation [12], evidence in mouse models demonstrates that autophagy inhibition can limit the growth of established tumors and improve the response to cancer therapeutics [13, 14]. Targeting autophagy is becoming an attractive strategy for cancer treatment. However, an atlas of autophagy in human primary cancer tissue is still lacking. The autophagic properties of cancer cells at different locations in human primary cancer tissues remain undefined. As a biological process responding to the intracellular and environmental changes, autophagy should be highly different in every single cancer cells exposed to different environments and exhibiting different cellular behaviors [15, 16]. Understanding the autophagic state in cancer cells with different behaviors will facilitate the development of therapeutic strategies involving autophagy targeting.

In this report, we used toluidine blue-staining-directed transmission electron microscopy (TEM) to evaluate autophagic flux in cancer cells at different locations in human primary lung cancer tissues. We also developed an *in vitro* live cell autophagy monitoring system to assess the change of autophagy in cancer cells during the invading process. Our studies demonstrated that cancer cells at the leading edge exhibited higher autophagic flux than cancer cells inside the tumor body, providing an *in vivo* evidence for understanding dynamic and functional autophagy process in cancer.

## RESULTS

### Cancer cells at the invasive front exhibit increased autophagic flux

**Figure 1:**
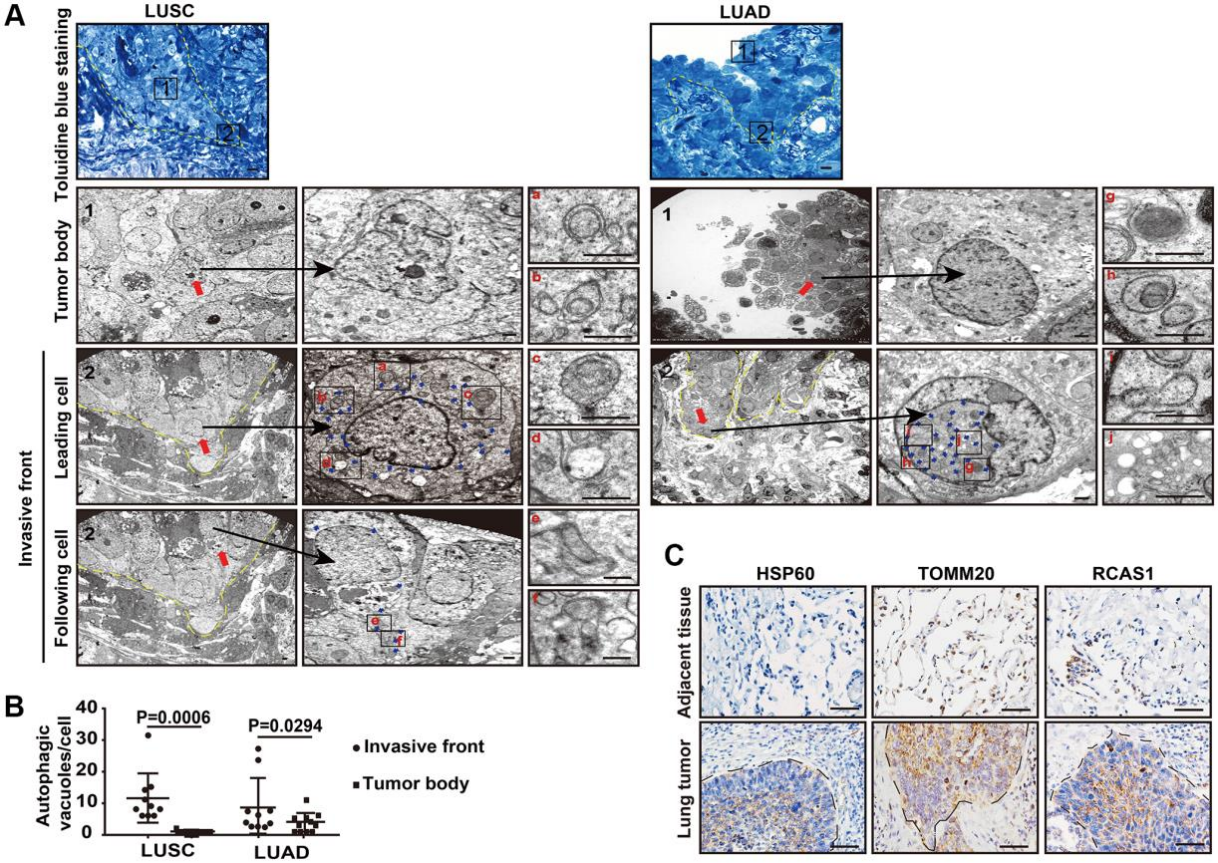
**The autophagic flux is increased at the tumor invasive front.** (**A**) Toluidine blue staining of the LUSC and LUAD for TEM (left panel). The TEM images show the autophagic vacuoles (blue arrows) in tumor cells located at the invasive front and inside the tumor body of LUSC and LUAD (right panel). Dashed line, tumor-stroma border. Red arrows, leader cells and following cells in invasive front and cells inside the tumor body. The enlarged images show autophagic vacuoles, including phagophores (a and g), autophagosomes (b and h), amphisomes (c, e, f and i) and autolysosomes (d and j). Scale bars, 50 μm for toluidine blue staining and 1 μm for TEM. (**B**) The quantification of the autophagic vacuoles per cell located at the tumor invasive front and inside the tumor body (*P* = 0.0006 in LUSC, *P* = 0.0294 in LUAD). Error bars, means ± SEM for 10 cells in a representative experiment. (**C**) Representative immunohistochemistry (IHC) images of HSP60, TOMM20 and RSCA1 in LUSC specimens and tumor-adjacent tissues. Scale bars, 50 μm.

We studied autophagic flux in cancer cells at different locations in human primary non-small cell lung cancers (NSCLCs) using ultrathin-section TEM. To precisely locate the invasive front in lung cancer tissues, we prescreened resin-embedded semi-thick sections of primary human lung cancer tissues, including two squamous lung cancers (LUSC) and two lung adenocarcinomas (LUAD), by toluidine blue staining, and the invasive front region was subjected to further TEM analysis (Figure 1A). Under TEM, we observed a large number of autophagy-related structures representing different stages of autophagy, such as phagophores, autophagosomes, amphisomes, and autolysosomes in the cells at the invasive front but not in cells inside the tumor body in both LUSC tissues and LUAD tissues (Figure 1A, 1B), suggesting that cancer cells at the invasive front may undergo very fast autophagic recycling.

**Figure 2:**
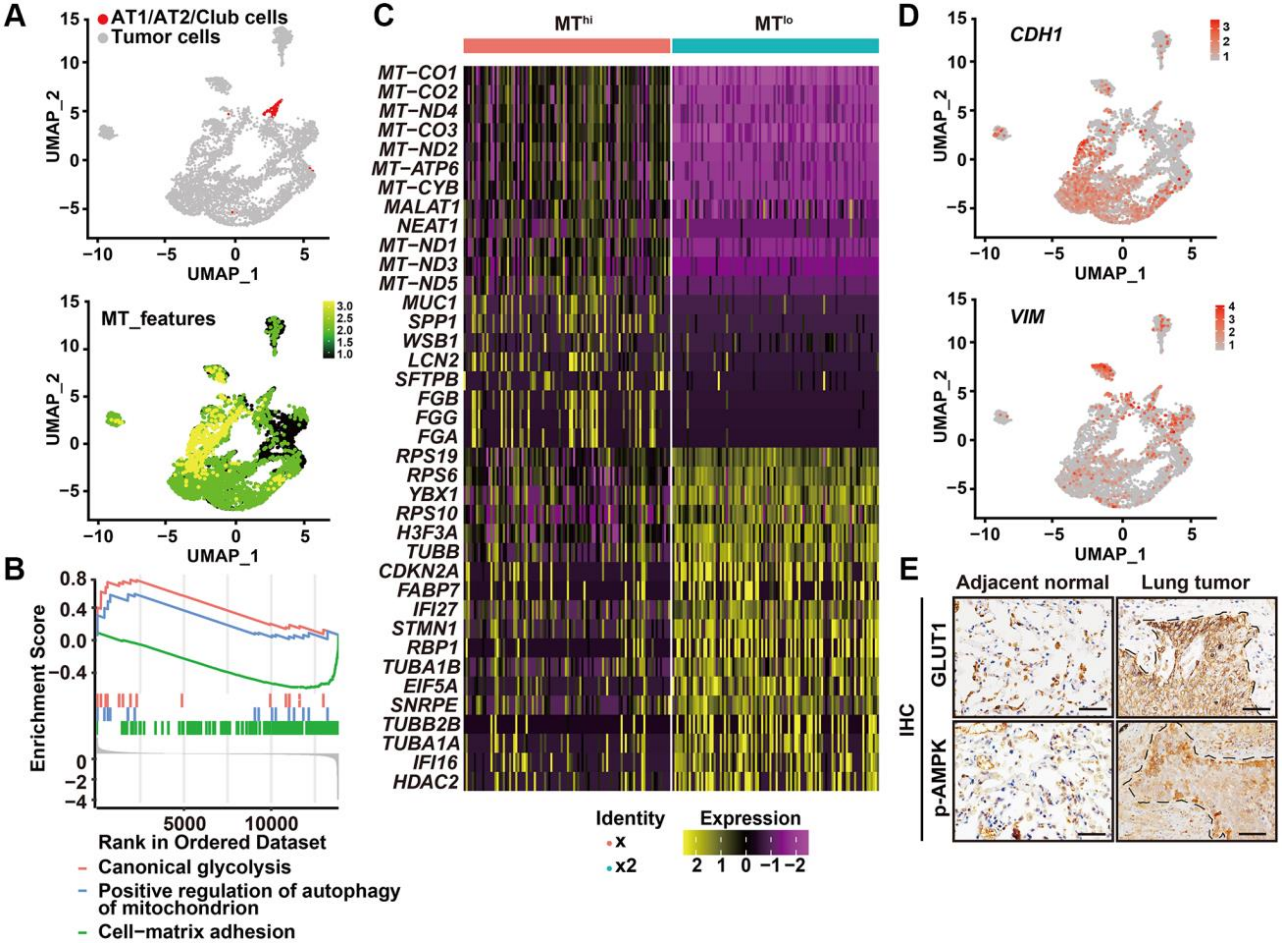
**High autophagic cancer cells exhibited low MT-gene signature.** (**A**) After removing the immune, endothelial and fibroblast cells, and re-clustering the remaining cells, predicted normal epithelial cell types, including AT1 (alveolar type I) cells, AT2 (alveolar type II) cells and club cells, based on gene expression were labeled (upper panel), and visualization of MT-gene signature scores (*MT-ATP8*, *MT-ATP6*, *MT-CO1*, *MT-CO2*, *MT-CO3*, *MY-CYB*, *MT-ND1*, *MT-ND2*, *MT-ND3*, *MT-ND4*, *MT-ND4L*, *MT-ND5*, *MT-ND6*) of malignant cells in 6 primary LUADs (lower panel). (**B**) GSEA analysis was performed to further screen the significant pathway between MT^lo^ and MT^hi^ cancer cells. (**C**) Heatmap showing the expression level of the top 20 differentially expressed genes for MT^lo^ and MT^hi^ clusters. (**D**) UMAP visualization of *CDH1* and *VIM* of malignant cells in 6 primary LUADs. (**E**) IHC staining of phosphorylated AMPK (p-AMPK) and GLUT1 in tumor-adjacent tissue and LUSC tissue. Dashed line, tumor-stroma border. Scale bars, 50 μm.

Interestingly, in these high autophagic cancer cells, less organelles including mitochondria, endoplasmic reticulum, or Golgi apparatus were observed (Figure 1A, 1B). Consistently, the heat shock protein 60 (HSP60), translocase of outer mitochondrial membrane 20 homologs (TOMM20) and the Golgi protein receptor binding cancer antigen expressed on SiSo cells (RCAS1) were less expressed at the invasive front, compared with the strong IHC signal of these proteins in the intratumoral cells (Figure 1C). Next, we evaluated the cellular functions by assessing the single-cell RNA-seq data of human primary lung cancers based on expression of mitochondria genes. We analyzed single-cell RNA-seq of six human primary LUADs including two stage I, three stage II and one stage III tumors in ArrayExpress under accessions E-MTAB-6149 and E-MTAB-6653 using the uniform manifold approximation and projection (UMAP) [17]. We removed the immune cells that expressed *PTPRC/*CD45, endothelial cells that expressed *PECAM1/*CD31, and fibroblast cells (Supplementary Figure 1) and regrouped the remaining cells using UMAP (Figure 2A, upper panel). Based on expression of mitochondria genes, cancer cells were divided into MT^lo^ subgroup (lacking detectable expression of the mitochondria genes) and MT^hi^ subgroup (Figure 2A, lower panel and Supplementary Figure 2). Compared to MT^hi^ cancer cells, MT^lo^ cancer cells exhibited upregulated mitophagy and glycolytic pathways, as expected, but downregulated cell-matrix adhesion pathway (Figure 2B). In addition, MT^lo^ cancer cells expressed higher levels of *SMTN1*, *TUBB*, *TUBA1A*, and *TUBA1B* genes involved in microtubule turnover which contributes to cellular processes such as intracellular transport, cell division and migration, than MT^hi^ cancer cells did [18] (Figure 2C). Also, MT^lo^ cells expressed high level of *VIM* but lack *CDH1* (Figure 2D). These transcriptomic features suggest that the high autophagic MT^lo^ cancer cells may reside in a mesenchymal state and exhibit reduced substrate-adherence while increased migration, and glycolysis. Consistently, cell surface expression of GLUT1 and phosphorylated AMPK (a protein that activate autophagy) were predominantly observed in cancer cells at the invasive front in immunostaining (Figure 2E). These collective results indicate that in human primary lung cancer tissues, invading cancer cells at the invasive front exhibit higher level of autophagic flux than cancer cells inside the tumor body.

### Autophagic flux is elevated in invading cancer cells *in vitro*

**Figure 3:**
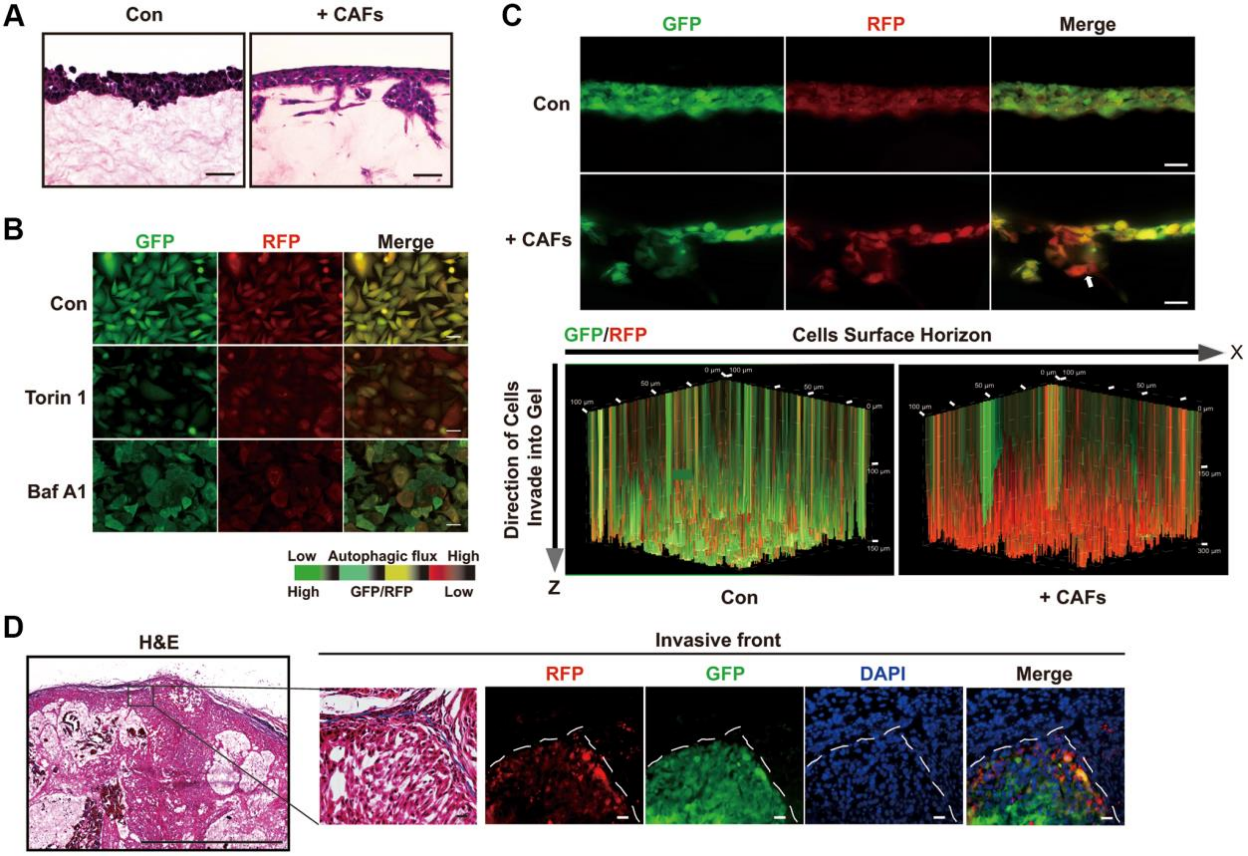
**Autophagic degradation and recycling are elevated during the invading process.** (**A**) H&E stained sections of 3D organotypic coculture gels. Scale bars, 50 μm. (**B**) The GFP/RFP ratio in single A549 cell colonies stable expressing the GFP-LC3-RFP-LC3ΔG cassette was determined under the indicated treatment. Scale bars, 200 μm. (**C**) Fluorescent images indicating GFP and RFP of A549 cells expressing GFP-LC3-RFP-LC3ΔG in cryosections of paraformaldehyde-fixed organotypic gels (upper panel) and GFP and RFP in a 3D volume rendering of live A549 cells expressing GFP-LC3-RFP-LC3ΔG (lower panel). White arrow, invasive front of A549 cells in the 3D organotypic coculture system. These fluorescent images of the gels in the 3D organotypic coculture system are representative of three independent experiments. Scale bars, 200 μm. The increments on the Z-axis indicate invasion distance (μm). (**D**) Representative images of H&E staining and GFP/RFP fluorescence in subcutaneous tumors formed by GFP-LC3-RFP-LC3ΔG-expressing A549 cells. Dashed line, tumor-stroma border. Scale bar, 200 μm for H&E staining and 50 μm for fluorescence images.

Then, we sought to develop an *in vitro* system to monitor autophagic flux in the invading cancer cells. To do this, we combined a live cell autophagy monitoring system with a three-dimensional (3D) organotypic invasion coculture system. Knowing that paracrine interaction between human cancer-associated fibroblasts (CAFs) and malignant cells promotes invasion of malignant cells at the tumor-stroma border [1], we seeded lung adenocarcinoma cells A549 on the surface of a dense gel composed of collagen I and Matrigel, which closely mimics the tumor-associated microenvironment, with or without human CAFs embedded in the gels. As expected, A549 cells were able to invade the gel only when CAFs were embedded. In the absence of embedded CAFs, A549 cells grew only on the top of the gel and could not invade it (Figure 3A).

To monitor the autophagic flux in this model, we transduced the fluorescent probe GFP-LC3-RFP-LC3ΔG into A549 cells to assess the formation and degradation of LC3 puncta [19]. Upon autophagy activation, this probe is cleaved by endogenous ATG4 proteases into equimolar amounts of GFP-LC3 and RFP-LC3ΔG. GFP-LC3 is degraded by autophagic process, while RFP-LC3ΔG remains in the cytosol, serving as an internal control. Thus, the GFP/RFP signal ratio correlates inversely with autophagic activity. As expected, the autophagy inducer torin1 significantly decreased the GFP/RFP ratio, whereas the autophagy inhibitor bafilomycin A1 (Baf A1) increased this ratio (Figure 3B).

Next, we monitored intact autophagic flux in live cells in the 3D organotypic coculture invasion system under a laser scan confocal microscope. A549 cells did not invade and showed substantial cytosol yellow fluorescence in the organotypic gels without embedded CAFs (Figure 3C, upper panel). In comparison with A549 cells grown in the absence of CAFs, A549 cells formed a bulky invasive front in coculture with CAFs, with the GFP/RFP ratio gradually decreasing along the Z-axis from the tumor-stroma border in the direction of invasion (Figure 3C, upper panel). Additionally, as the scanning depth was increased, the invasion distance of the tumor cells increased, resulting in a decreased GFP/RFP ratio and suggesting enhanced autophagic flux in the invading cancer cells (Figure 3C, lower panel). Confocal imaging of cryosections of paraformaldehyde-fixed organotypic gels confirmed the reduced GFP/RFP ratio in the invading cancer cells (Figure 3C). Consistent with these observations, tumors formed from subcutaneous engraftments of GFP-LC3-RFP-LC3ΔG-expressing A549 cells exhibited a lower GFP/RFP ratio in the invasive front at the tumor-stroma border than inside the tumor body (Figure 3D). Taken together, the results of these *in vitro* and *in vivo* assays indicate that autophagy is increased in cancer cells at the invasive front, consistent with the results of our above TEM in human primary lung cancer tissues.

### Elevated autophagic flux is required during the invasion process and potentiates cells invasiveness

**Figure 4:**
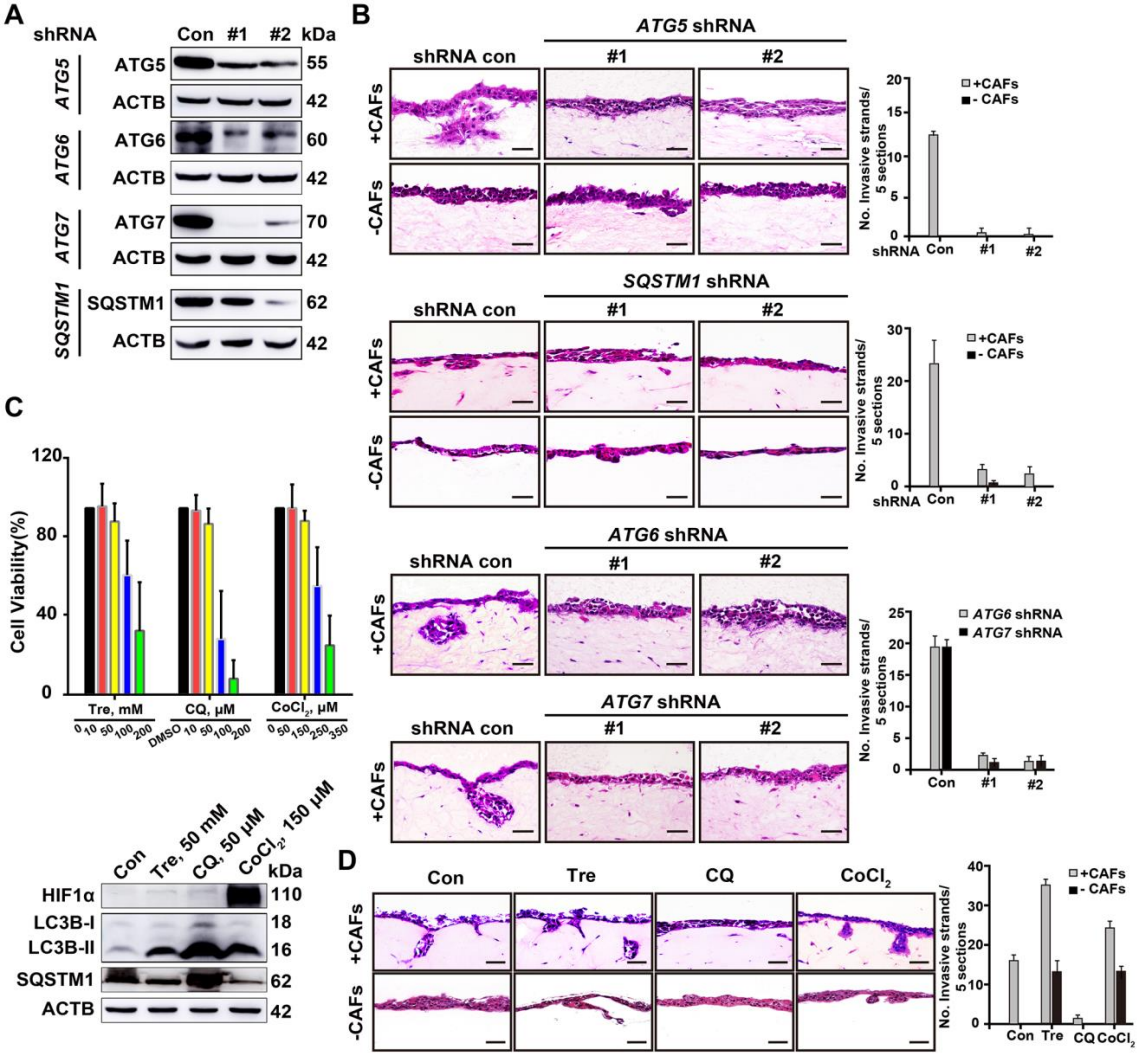
**Deficient autophagy blocks cell invasion into organotypic gels.** (**A**) The knockdown efficiency of shRNAs for autophagy-related proteins, including ATG5, ATG6, ATG7 and SQSTM1, was assessed by Western blotting. (**B**) Representative H&E-stained sections of *ATG5*-, *ATG6*-, *ATG7*- and *SQSTM1*-depleted A549 cells in the 3D organotypic coculture invasion system with or without human CAFs. The experiment was performed in triplicate. Scale bars, 50 μm. (**C**) The viability of A549 cells was evaluated by a CCK8 assay after 72 h of treatment with Tre, CQ, DMSO (vehicle control), or CoCl_2_ at the indicated concentration (upper panel). Error bars, means ± SD of a representative set of triplicate experiments. Immunoblots of HIF1α, LC3B, and SQSTM1 with the indicated treatment for 10 h, ACTB used as a loading control (lower panel). (**D**) The invasion capability of A549 cells was evaluated in the 3D organotypic coculture invasion system on gels with or without CAFs under treatment with DMSO, Tre (50 mM), CQ (50 μM), or CoCl_2_ (150 μM) for 10 days. The experiment was performed in triplicate. Scale bars, 50 μm. The number of invasive strands was quantified in 5 H&E-stained sections of each organotypic coculture gels. Error bars, means ± SD of a representative set of triplicate experiments.

Autophagy-related genes (ATGs) including *ATG5*, *ATG6* (also known as *BECN1*), *ATG7*, and *SQSTM1* are key components involved in specific steps during autophagosome biogenesis [20]. To further evaluate the impact of autophagy on the invasive ability of cancer cells, we depleted these *ATGs* by shRNA-mediated knockdown and tested the changes in invasion using the 3D organotypic coculture invasion system. Inhibition of *ATG6*, *ATG5*, *ATG7*, or *Sequestosome 1 (SQSTM1)/p62* blocks the nucleation of the autophagosome, the expansion and completion of the autophagosome membrane, and the maturation and degradation of the autophagosome membrane, respectively [16]. Interestingly, depletion of these *ATGs* profoundly attenuated A549 cell invasion in the presence of CAFs in the 3D organotypic coculture invasion assay (Figure 4A, 4B). Therefore, impairment of autophagic initiation or autophagic degradation in lung cancer cells decreased invasion.

Next, we modulated autophagic flux in the A549 cells using trehalose (Tre, an autophagy inducer) [21], CoCl_2_ (a hypoxia-dependent autophagy inducer) [22], or CQ (an autophagic degradation inhibitor) [23] to assess cell invasion in the same model, respectively. We treated the A549 cells with Tre, CQ, or CoCl_2_ at different concentrations and selected a dose at which cell viability was not affected but autophagic flux was changed, as evaluated by SQSTM1 accumulation and microtubule-associated protein 1 light chain 3 B (MAPLC3B/LC3B)-II/LC3B-I ratio (Figure 4C). Tre (50 mM) treatment and hypoxic condition (CoCl_2_, 150 μM) promoted more cells invading from the tumor-stroma border into the CAFs-containing gels, but CQ (50 μM) treatment completely inhibited this invasion (Figure 4D). Notably, even in the absence of CAFs, the elevated autophagic flux proficiently endowed A549 cells with invasive ability, whereas CQ treatment did not (Figure 4D). These results indicate that a high level of autophagic flux is required for cells invasion and is sufficient for the invasion of cancer cells in the absence of an interaction with stromal cells.

### Increased SQSTM1-LC3B interaction at the tumor invasive front is correlated with poor prognosis for NSCLC patients

**Figure 5:**
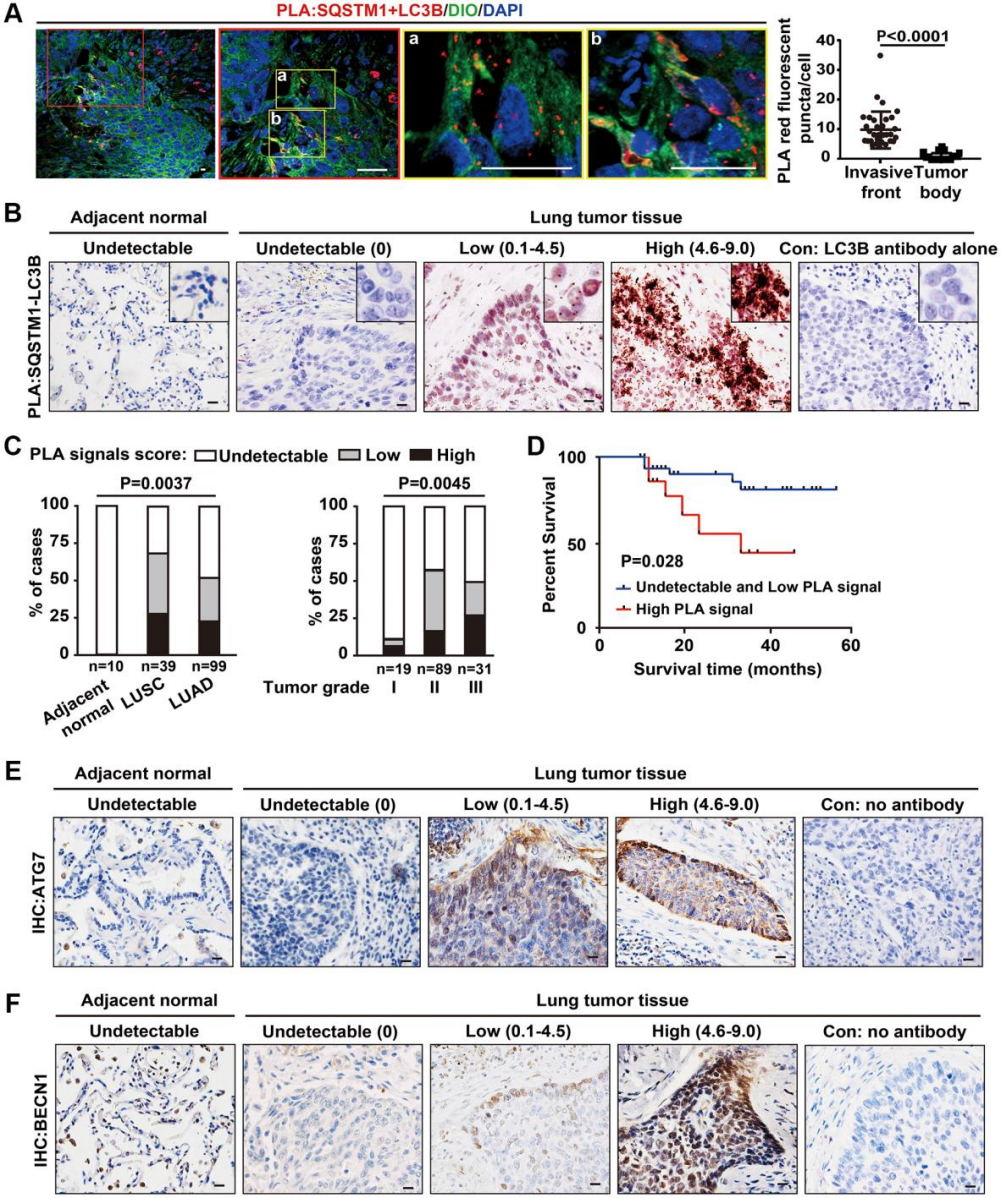
**Increased SQSTM1-LC3B interaction at the tumor invasive front is correlated with poor outcome in NSCLC patients.** (**A**) Endogenous physical interactions between SQSTM1 and LC3B in LUSC sections were detected by *in situ* PLA (indicated by red fluorescent puncta) and visualized by laser scan confocal microscopy. Membrane structures were counterstained with a DIO probe (green). The enlarged regions show positive PLA signals of SQSTM1-LC3B protein complexes at the tumor invasive front (a-b). Scale bars, 50 μm. The scatter diagram of the PLA results shows the number of red fluorescent puncta per cell at the tumor invasive front and inside the tumor body (*P* < 0.0001). Error bars, means ± SEM for 30 cells in a representative experiment. (**B**) Representative images of *in situ* bright-field PLA of SQSTM1-LC3B protein complexes in NSCLC specimens and tumor-adjacent tissues, counterstained by Hematoxylin. Scale bars, 50 μm. (**C**) *in situ* PLA of SQSTM1-LC3B protein interaction was performed on tumor-adjacent lung tissues (*n* = 10), LUSC (*n* = 39), and LUAD (*n* = 99) specimens. The frequency of patient with undetectable (0), low (< 4.5), or high (4.6–9.0) PLA signal scores stratified by IHC-defined lung cancer subtype (ANOVA, *P* = 0.003) (left panel) or by NSCLC grade (ANOVA, *P* = 0.0045) (right panel). (**D**) Survival rates in 64 of the 148 NSCLC patients determined by Kaplan-Meier analysis were compared between patients with undetectable and low (PLA signals score ≤ 4.5, *n* = 48, blue line) and high (PLA signals score > 4.6, *n* = 16, red line) PLA signal scores for the SQSTM1-LC3B interaction. Three-year survival rates were 85% (undetectable and low PLA signal scores of SQSTM1-LC3B interaction) versus 33% (high PLA signal scores of SQSTM1-LC3B interaction; *P* = 0.028). (**E** and **F**), Representative IHC images of ATG7 and BECN1 in NSCLC specimens and tumor-adjacent tissues. Scale bars, 50 μm.

The physical interaction between SQSTM1 and LC3B is essential for autophagosome formation [24]. To accurately evaluate the location at which the SQSTM1/LC3B interactions occur, we used highly sensitive *in situ* proximity ligation assay (PLA) to assess the dynamic autophagic flux in cancer cells at different locations in the corresponding human lung cancer tissue sections, counterstained the tumor tissue sections with DIO, a dye to label membrane. Using this approach, we observed significantly more positive SQSTM1/LC3B interactive signals, most of which located in the cytosol, in cancer cells at the invasive front than in cancer cells inside the tumor body (Figure 5A).

To investigate the correlation of autophagy at the invasive front with patient prognosis, we studied the interaction of SQSTM1 and LC3B in 148 NSCLC specimens, including 39 LUSC and 99 LUAD specimens, and in 10 tumor-adjacent lung tissues using bright-field PLA. No PLA signal of SQSTM1/LC3B interaction was detected in epithelial cells in tumor-adjacent lung tissues, indicating a relatively low autophagic flux (Figure 5B). The PLA signal of SQSTM1/LC3B interaction was found mainly in the cytoplasm of NSCLCs at the invasive front, consistent with the results of fluorescent PLA shown in Figure 5A, representing the high levels of autophagic flux at tumor invasive front (Figure 5B), consistent with our previous observations under TEM.

Quantification of PLA signals based on the intensity of PLA puncta and percentage of SQSTM1/LC3B complex-positive tumor cells revealed higher levels of SQSTM1/LC3B protein complexes in LUSCs than in LUADs (Figure 5C, left panel). In addition, a higher PLA signal score of the SQSTM1/LC3B interaction was associated with an advanced stage of NSCLC (Figure 5C, right panel). To assess the prognostic significance of SQSTM1/LC3B protein complexes, we examined their levels in resected NSCLC tissues from 64 subjects with known clinical outcomes. Patients with tumors showing undetectable and low PLA signal scores for SQSTM1/LC3B interaction (PLA signal score ≤ 4.5, *n* = 48) had longer disease-free survival time than patients whose tumors had high PLA signal score (PLA signal score > 4.5, *n* = 16), with three-year survival rates of 85% for those with low PLA signal score for the SQSTM1/LC3B interaction and 33% for those with high PLA signal scores (Figure 5D). These results indicate that the abundance of SQTM1/LC3B complexes is increased at the in invasive front of lung cancers and that this high abundance correlates with poor prognosis in human lung cancer. Consistently, we also noticed that the expression of ATG7 and BECN1 were predominantly located at the tumor edge rather than tumor nest by IHC in these tumor tissues (Figure 5E, 5F). Thus, the detection of the SQTM1/LC3B complex may have a value for predicting cancer cell invasiveness around the invasive front in clinical cancer samples. However, based on the dynamic autophagy changes in cancer cells, the use of autophagy-targeting therapies in cancer patients still require a much deeper understanding of autophagic processes that mediate cancer cell behaviors.

## DISCUSSION

Human lung cancers are widely known highly heterogeneous as revealed by transcriptomic, genomic, epigenomic, and proteomic profiles analysis [7, 25, 26]. In this study, we provide the *in vivo* evidence showing that cancer cells at the invasive front exhibit a higher level of autophagic flux than cancer cells inside the tumor body do. It is noteworthy that the high autophagic cancer cells at the invasive front contain very few other organelles such as mitochondria, Golgi, and ERs. This suggests that these high autophagic cancer cells do not rely on mitochondrial metabolism to generate ATP. Instead, they may adapt to glycolysis to meet ATP demand, which subsequently leads to lactate production, micro-environmental acidosis, and metastasis [27, 28]. This scenario is supported by our analysis of transcriptional profiling of those single lung cancer cells that lack expression of mitochondria encoded genes, showing the increased glycolytic and migratory profiles. Our findings are consistent with the observation that tumor cells exhibit rapid glucose uptake and lactate secretion, as well as suppression of mitochondrial metabolism [29].

It has been noticed that autophagy is dysregulated in many cancers and dysregulation of autophagy can be both tumor promotional and tumor suppressive, depending on the cellular context [16]. In established tumors, autolysosomal degradation promotes their ability to adapt to limiting nutrient supplies, therefore maintaining cellular aggressiveness [30, 31]. Although it is well established that autophagic inhibition represses cancer invasion, a full view of the diversity of autophagic states in human primary lung cancer tissues remain less certain. Our data suggest that a rapid autophagic recycling process might also be needed for mitochondria downregulation. The supporting data include 1) higher levels of cytoplasmic LC3/SQSTM1 complexes by *in situ* PLA, 2) the increased number of autophagic vesicles at the invasive front of human primary NSCLC tissue, and 3) the enhanced autophagic flux in the live invading cancer cells of the 3D organotypic cocultures. Indeed, the invading cancer cells locate at the tumor-stroma border and interact with a complex microenvironment containing extracellular matrix that provides active or passive mechanical cues [4], such as fibroblasts that secret various growth factors, cytokines, chemokines and proteinases [32], and leaky blood vessels that change the pH and oxygen supplement of the microenvironment [33]. We also examined hypoxic status by immunostaining for HIF1α in lung cancer tissue sections. Cancer cells at the invasive strand and cancer cells inside the tumor body, which exhibited different cytoplasmic/dot-like SQSTM1 staining, showed similar HIF1α staining intensity (data not shown). Thus, the elevated autophagic flux in invading cancer cells may not due to the limited nutrient or oxygen supplement.

In our 3D organotypic cocultures, cancer cells could not invade without CAFs embedded in the gel. The bottom layer of cancer cells directly interact with the gel which contains various soluble signals but does not show enhanced autophagic flux. With embedded CAFs in the gel, cancer cells started to invade. Invading cancer cells encounter CAFs and a physical force dictated by extracellular matrix besides those soluble signals in the gel. Given that both invading cancer cells and non-invading cancer cells interact with those soluble signals, it is possible that either one or both of physical force and CAFs caused enhanced autophagic flux. Mechanical cues elicit biochemical signals and regulate cell function and behavior through mechanotransduction [34]. For example, YAP/TAZ, downstream effectors of extracellular matrix dependent cellular mechanotransduction, can regulate autophagic flux in pancreatic and mammary cancer cells [35]. Nevertheless, tumor cell plasticity is required in transition states including epithelial-mesenchymal transition (EMT) and mesenchymal-epithelial transition (MET), which may vary based on the tumor type, the state of dissemination, and the context of tumor metastasis [36]. The invasive front cancer cells express the mesenchymal marker vimentin but lack the epithelial marker E-cadherin, and exhibit mesenchymal attributes [9]. Further studies and better models that cancer cells can imitate *in vivo* invasion are needed to illustrate whether rigidity of extracellular matrix is indispensable to the regulation of autophagic flux.

Since a higher level of autophagic flux is required and sufficient for cancer cells to invade into the surrounding microenvironment, inhibition of autophagic flux through knockdown of autophagy proteins or the use of conventional small inhibitory molecules suppressed the invasion of cancer cells. In contrast, inducing autophagy with an autophagic stimulator such as trehalose or CoCl_2_ endows cancer cells with invasive capability in a 3D organotypic coculture system. Furthermore, suppression of autophagy inhibits cellular invasion but not polarization, consistent with the previous reports [37–39]. However, suppression of autophagy can also promote cancer cell invasion [40–42], dictating the context-dependent autophagy on metastatic progression. The functional roles of tumor cells as well stromal cells within tumor microenvironment are extremely complex and are critical to diverse aspects of tumor biology [43].

In summary, our data showed that invading cancer cells require increased autophagic flux to migrate and invade at the invasive front. Targeting autophagy at the invasive front in the tumor-stroma border may serve as an adjuvant therapeutic strategy to improve treatment efficacy.

## MATERIALS AND METHODS

### Human samples

Human lung cancer tissues were collected at Tianjin Medical University Cancer Hospital. The use of all human lung cancer tissues was approved by the Institutional Review Board of Tianjin Medical University. Informed consent was obtained from all patients, and samples were deidentified prior to analysis.

### TEM

Samples from human primary lung cancer tissues were fixed in a fixative solution containing 6% glutaraldehyde followed by post-fixed with 2% osmium tetroxide (Sigma-Aldrich, 75632), thereafter were dehydrated in ascending ethanol solutions and absolute acetone, immersed in 50% Epon812 (Electron Microscopy Sciences, 14120) in acetone and embedded. The semi-thick sections were subjected to toluidine blue staining to identify the tumor invasive front and tumor body. The serial ultra-thin sections of invasive front and tumor body that identified by toluidine blue staining subsequently stained with uranyl acetate and lead citrate. Digital TEM images were acquired from thin sections using a HITACHI HT7700 transmission electron microscope (Hitachi High-Tech, Japan).

### Cells, lentiviral transduction, and chemicals

A549 cells were purchased from ATCC and maintained in ATCC recommended media: RPMI1640 (Thermo Fisher Scientific) with 10% fetal bovine serum, 100 U of penicillin/mL, 100 μg of streptomycin/mL (Thermo Fisher Scientific). All experiments were performed within 1 month after thawing early-passage cells. A549 cells were tested negative for mycoplasma and were authenticated in April 2017. DNA purified from A549 cell lines were tested by the short tandem repeat analysis method using Promega PowerPlex 1.2 analysis system (Genewiz Inc.). Data were analyzed using GeneMapper4.0 software and then compared with the ATCC databases for reference matching. Human cancer-associated fibroblasts were isolated from primary lung tumors by physical fragmentation and 2 mg/ml collagenase (Sigma-Aldrich, C2139) digestion and maintained in DMEM supplemented with 10% FBS, 100 U of penicillin/mL, 100 μg of streptomycin/mL, 1% OriCellTM ITS (Cyagen) and 2 mM GlutaMAXTM-I (Thermo Fisher Scientific). For lentiviral transduction, Phoenix-293T cells were seeded in 100-mm plates and co-transfected with the target constructs and the packaging plasmids pMD2.VSVG, pMDLg/pRRE, and pRSV-REV. A549 cells were allowed to viral infect in 35-mm plates for 8 h and recover for 24 h prior to the further procedure. Single cell clones were developed for all the following assays. The autophagy inducers and inhibitors used in this study were purchased from Sigma-Aldrich: torin1 (475991), trehalose (Tre, T9531), Cobalt chloride hexahydrate (CoCl_2_, V900021), bafilomycin A1 (Baf A1, 196000), chloroquine (CQ, C6628). GFP-LC3-RFP-LC3ΔG probe was purchased from Addgene, 84572. The DNA sequences for shRNA knockdown assay are listed in Table 1. All primary antibodies for this study are listed in Table 2.

**Table 1:** shRNA sequences used in this study.

**shRNA target sequence (5′–3′)**	**Efficiency (%)**
*ATG5* (human, NM_00449)	
#1 GCAACTCTGGATGGGATTG	70
#2 AGTGAACATCTGAGCTACC	80
*ATG7* (human, NM_006395)	
#1 GCCTGCTGAGGAGCTCTCCA	80
#2 CCCAGCTATTGGAACACTGTA	75
*ATG6/NECN1* (human, NM_003766)	
#1 ACAGGAGCTGGAAGATGTGGAA	85
#2 AGCCAATAAGATGGGTCTGAAA	80
*SQSTM1/p62* (human, NM_003900)	
#1 GGAGCACGGAGGGAAAAGATT	70
#2 GTGACGAGGAATTGACAATTT	84
*Firefly luciferase* (con)	
CGTACGCGGAATACTTCGATT	Irrelevant control

**Table 2:** Primary antibodies used in this study.

**Primary Antibody**	**Dilution Ratio**		**Supplier**
	**IHC/PLA**	**WB**	
ATG5	1:100	1:5,000	Abcam, ab109490
ACTB		1:3,000	Sigma-Aldrich, A-3853
ATG7	1:100	1:1,0000	Abcam, ab52472
BECN1	1:100		BIOSS, bs-1353R
HSP60	1:100		Santa Cruz Biotechnology, sc-376261
RSCA1	1:100		Abcam, ab16568
TOMM20	1:100		Abcam, ab186735
LC3B	1:100/1:100	1:2,000	Sigma-Aldrich, L7543
SQSTM1/p62	1:400/1:100	1:1,000	Abcam, ab56416
HIF1a	1:400	1:3,000	Abcam, ab51608
ATG6/BECN1		1:2,000	Cell Signaling Technology, 3738
p-AMPK	1:100		Cell Signaling Technology, 2535
GLUT1	1:100		Abcam, ab115730

### 3D organotypic coculture

The 3D organotypic coculture invasion assays were performed as follows: fibrillar rat-tail collagen I (Corning; 354249) and growth factor-reduced Matrigel (BD Biosciences; 356230) were mixed to generate a mixture gel containing collagen at 4 mg/ml and Matrigel at 2 mg/ml. Neutralized mixture gel 600 μL/well was then aliquoted onto a 0.4 μm polyester membrane (Corning Costar; 34818034). For assays involving the human cancer-associated fibroblasts (CAFs), 5 × 10^5^ human CAFs were embedded in 1 ml mixture. The 3D organotypic coculture invasion gel with or without human CAFs was allowed to polymerize at 37 °C for about 1 h, after that 5 × 10^5^ A549 cells were plated on the top of gel. The gel was fed from underneath with complete medium supplemented 1% insulin-transferrin-selenium (Thermo Fisher Scientific; 51500-056) and 2 mM GlutaMAXTM-I (Thermo Fisher Scientific). Medium was changed daily. Tumor cells invade into gels over a period of 8 to 10 days. Gel was fixed using 4% paraformaldehyde plus 1% glutaraldehyde in PBS and embedded in paraffin or optimal cutting temperature compound (SAKURA, T4583). 5 μm paraffin sections were subjected to Hematoxylin and Eosin (H&E) staining and 10 μm cryo-sections were visualized by confocal microscope.

### *in situ* PLA

All the tumor tissues from the patients received standard surgical excision, after that, tumor tissues were fixed in 4% paraformaldehyde in phosphate-buffered saline (PBS, pH 7.4; Invitrogen, 10010023) for 24 h, embedded in paraffin, sectioned at 5 μm. *in situ* PLA was performed to visualize protein-protein interactions in lung cancer tissues. Briefly, paraffin-embedded tissues were deparaffinized and rehydrated using standard methods. After heat-mediated antigen retrieval in citrate buffer (10 mM citric acid, pH 6.0) at 100°C for 40 min, sections were permeabilized with PBS containing 0.5% Triton X-100 (Sigma-Aldrich, SLCD3237) for 30 min, subsequently blocked in Blocking Solution (Sigma-Aldrich, DUO92004) at 37°C for 1 h and incubated with primary antibodies overnight at 4°C. On the following day, sections were washed twice with washing buffer (Sigma-Aldrich, DUO82049), and incubated with PLA probes in a ratio of 1:10 in antibody diluent for 1 h at 37°C. The sections were then incubated with ligation solution at 37°C for 30 min and subsequently with amplification solution at 37°C for 120 min. For fluorescent PLA, the tumor tissue sections were counterstained with 3, 3’-dioctadecyloxacarbocyanine perchlorate (DIO, Beyotime, C1038), a dye to label membrane or mounting medium (Origene, ZLI-9557) together with DAPI for 10 min. Cell images were captured with a confocal microscope (Carl Zeiss, Axio Imager A2, Germany). For bright-field PLA, sections were incubated with Detection Solution (Sigma-Aldrich, DUO92012B) at room temperature for 60 min and then developed with Substrate Solution (Sigma-Aldrich, DUO92012B) for 5–10 min. Nuclear was stained with Nuclear Stain (Sigma-Aldrich, DUO92012B) for 5 min.

For evaluation of PLA signal score of SQSTM1-LC3B complex in tumor tissue array, we defined the PLA puncta expression patterns into an intensity score from 0-3 for quantitative evaluation as follows: score 0, no puncta or barely visible puncta in <5% of the cells; score 1, detectable puncta in 5–25% of cells; score 2, readily detectable puncta in 25–75% of cells; score 3, puncta in >75% of cells. The number of cytoplasmic PLA puncta was assessed as absent (score 0), weak (score 1), moderate (score 2) or strong (score 3). Cytoplasmic PLA signal for SQSTM1-LC3B complex was classified as low and undetectable signal for a score of 0–4.5 and high signal for a score of 4.6–9.0.

### Single-cell RNA-seq data accessibility and processing

We accessed processed data of single-cell RNA-seq from ArrayExpress database (Accessions # E-MTAB-6149 and E-MTAB-6653) for our analysis [17]. To visualize the processed data from Cell Ranger analysis, we utilized Seurat suite version 3.1 to perform cell clustering. First, filtered gene-barcode matrix of the sample identified by Cell Ranger Count was inputted into Seurat. Low quality cells/dying cells were removed, and we retained the cells with properly molecular read count and gene number (nCount_RNA < 50000, nFeature_RNA < 6000, and nFeature_RNA >200) and with low mitochondrial transcript proportion (percent.mt < 25). Then we normalized and scaled the data, and then performed linear dimensional reduction by PCA (Principal components analysis) on the scaled data. Next, we clustered and visualized the cell distribution by UMAP (Uniform Manifold Approximation and Projection). By checking the know cell type markers, we well defined normal mesenchymal cells, epithelial cells and cancer cells. Then the cancer cell extraction and other further analysis were also performed by Seurat package and gene expression was visualized by UMAP.

### Single-cell RNA-seq differentially expressed (DE) gene identification

Tumor cells with low expression of 13 mitochondrial genome code genes were subset from the other tumor cells using “Subset” function and cells expressing both low and high mitochondrial genes were used for further analysis as two groups. DE genes were identified by “Find All Markers” command and top 20 genes of each group were visualized by heatmap on 100 randomly selected cells from each group by “downsample = 100”.

### Xenograft assay

2 × 10^7^ cells (1:1 of GFP-LC3-RFP-LC3ΔG-expressing A549 cells and CAFs) were suspended in 100 μL medium containing 50% Matrigel and were subcutaneously injected into 8-week-old female nude mice. 8 weeks after the inoculation, the mice were sacrificed and the tumors were dissected for further analysis. All animal procedures were approved by Animal Care and Use Committee at Tianjin Medical University and conform to the legal mandates and national guidelines for the care and maintenance of laboratory animals.

### Immunohistochemistry (IHC)

For IHC staining, antibodies were processed following the standard protocol. Briefly, deparaffinized and rehydrated using standard methods. After heat-mediated antigen retrieval in citrate buffer (10 mM citric acid, pH 6.0) at 100°C for 40 min, sections were blocked with a solution of 10% normal goat serum (NGS) in PBS at room temperature for 30 min. For primary antibody, an overnight incubation at 4°C was followed by 2 h incubation at room temperature with secondary antibodies (Thermo Fisher Scientific). Sections were then stained with the DAB (diaminobenzidine) substrate kit (ORIGENE, ZLI-9017), washed with deionized water and counterstained with Hematoxylin.

### Confocal microscopy

To obtain GFP and RFP fluorescence signals in the 3D organotypic coculture invasion system, gels was placed onto the glasses without fixing at the endpoint of the experiment. The Zst images were captured using a laser scanning confocal microscope immediately (Carl Zeiss, Axio Imager A2, Germany). In brief, serial scanning was performed at the XY level by moving the scanning depth under identical imaging conditions, including the same high voltage, offset and gain settings, same frame size, same pixel size, and same z-stack interval (5 μm) to acquire z-series images. During the process, RFP signals were uniformly strong irrespective of depth from the surface and accurately matched GFP signals. For 3D reconstructions, 14 (– CAFs) and 30 (+ CAFs) slices of 5 μm each from the whole 3D organotypic coculture invasion gels were fluorescently imaged, and the 3D volume render were performed by the extended depth of focus after stacking images along the Z-axis sequentially. Inserts in the z-axis represented the sum of z slices of 3D organotypic coculture invasion gels and illustrated the depth-distribution of fluorescence.

### Western blot

Protein lysates of cells were prepared using RIPA buffer (Thermo Fisher Scientific, 89900) and protein concentration was quantified using the BCA protein assay kit (Thermo Fisher Scientific, 23235) after sonication. Primary antibodies diluted in blocking buffer were incubated overnight at 4°C followed by a 2 h incubation of secondary goat-anti-rabbit IgG and goat-anti-mouse IgG antibodies (Thermo Fisher Scientific, 1:10,000) at room temperature. Membranes were visualized using the Amersham Imager 600 system (GE Healthcare, USA).

### CCK8 assay

Cell viability was examined using the Transdetect Cell Counting Kit (Transgen, Beijing, China) according to the manufacturer’s instructions. Briefly, A549 cells were grown in a 96-well plate for 24 h, followed by the treatment with DMSO, Tre, CQ or CoCl_2_ for the indicated time, respectively. To measure the cell viability, 10 μL of CCK-8 was added to each well for 2 h. Absorbance was measured at a wavelength of 450 nm.

### Statistical analysis

Our experimental data were summarized as the mean ± standard error or mean ± deviation and statistically analyzed using a *t*-test and ANOVA. Graphpad 7 was used for statistical analysis. High vs. low and undetectable PLA signal score of SQSTM1-LC3B protein interaction was statistically analyzed using the Kaplan-Meier curves and the log-rank test for the disease-free survival of lung cancer patients. A *P*-value of less than 0.05 was considered statistically significant.

## Supplementary Materials

Supplementary Figures

## References

[1] Puram SV, Tirosh I, Parikh AS, Patel AP, Yizhak K, Gillespie S, Rodman C, Luo CL, Mroz EA, Emerick KS, Deschler DG, Varvares MA, Mylvaganam R, et al. Single-Cell Transcriptomic Analysis of Primary and Metastatic Tumor Ecosystems in Head and Neck Cancer. Cell. 2017; 171:1611–24.e24. 10.1016/j.cell.2017.10.044 29198524 PMC5878932

[2] Schürch CM, Bhate SS, Barlow GL, Phillips DJ, Noti L, Zlobec I, Chu P, Black S, Demeter J, McIlwain DR, Kinoshita S, Samusik N, Goltsev Y, Nolan GP. Coordinated Cellular Neighborhoods Orchestrate Antitumoral Immunity at the Colorectal Cancer Invasive Front. Cell. 2020; 182:1341–59.e19. 10.1016/j.cell.2020.07.005 32763154 PMC7479520

[3] Gaggioli C, Hooper S, Hidalgo-Carcedo C, Grosse R, Marshall JF, Harrington K, Sahai E. Fibroblast-led collective invasion of carcinoma cells with differing roles for RhoGTPases in leading and following cells. Nat Cell Biol. 2007; 9:1392–400. 10.1038/ncb1658 18037882

[4] Kai F, Drain AP, Weaver VM. The Extracellular Matrix Modulates the Metastatic Journey. Dev Cell. 2019; 49:332–46. 10.1016/j.devcel.2019.03.026 31063753 PMC6527347

[5] McGranahan N, Swanton C. Biological and therapeutic impact of intratumor heterogeneity in cancer evolution. Cancer Cell. 2015; 27:15–26. 10.1016/j.ccell.2014.12.001 25584892

[6] Robertson-Tessi M, Gillies RJ, Gatenby RA, Anderson AR. Impact of metabolic heterogeneity on tumor growth, invasion, and treatment outcomes. Cancer Res. 2015; 75:1567–79. 10.1158/0008-5472.CAN-14-1428 25878146 PMC4421891

[7] Teixeira VH, Pipinikas CP, Pennycuick A, Lee-Six H, Chandrasekharan D, Beane J, Morris TJ, Karpathakis A, Feber A, Breeze CE, Ntolios P, Hynds RE, Falzon M, et al. Deciphering the genomic, epigenomic, and transcriptomic landscapes of pre-invasive lung cancer lesions. Nat Med. 2019; 25:517–25. 10.1038/s41591-018-0323-0 30664780 PMC7614970

[8] Lee CK, Jeong SH, Jang C, Bae H, Kim YH, Park I, Kim SK, Koh GY. Tumor metastasis to lymph nodes requires YAP-dependent metabolic adaptation. Science. 2019; 363:644–49. 10.1126/science.aav0173 30733421

[9] Du W, Xu X, Niu Q, Zhang X, Wei Y, Wang Z, Zhang W, Yan J, Ru Y, Fu Z, Li X, Jiang Y, Ma Z, et al. Spi-B-Mediated Silencing of Claudin-2 Promotes Early Dissemination of Lung Cancer Cells from Primary Tumors. Cancer Res. 2017; 77:4809–22. 10.1158/0008-5472.CAN-17-0020 28754672

[10] Ji AL, Rubin AJ, Thrane K, Jiang S, Reynolds DL, Meyers RM, Guo MG, George BM, Mollbrink A, Bergenstråhle J, Larsson L, Bai Y, Zhu B, et al. Multimodal Analysis of Composition and Spatial Architecture in Human Squamous Cell Carcinoma. Cell. 2020; 182:497–514.e22. 10.1016/j.cell.2020.05.039 32579974 PMC7391009

[11] Galluzzi L, Pietrocola F, Bravo-San Pedro JM, Amaravadi RK, Baehrecke EH, Cecconi F, Codogno P, Debnath J, Gewirtz DA, Karantza V, Kimmelman A, Kumar S, Levine B, et al. Autophagy in malignant transformation and cancer progression. EMBO J. 2015; 34:856–80. 10.15252/embj.201490784 25712477 PMC4388596

[12] Liu Z, Chen P, Gao H, Gu Y, Yang J, Peng H, Xu X, Wang H, Yang M, Liu X, Fan L, Chen S, Zhou J, et al. Ubiquitylation of autophagy receptor Optineurin by HACE1 activates selective autophagy for tumor suppression. Cancer Cell. 2014; 26:106–20. 10.1016/j.ccr.2014.05.015 25026213 PMC4166492

[13] Wen X, Klionsky DJ. At a glance: A history of autophagy and cancer. Semin Cancer Biol. 2020; 66:3–11. 10.1016/j.semcancer.2019.11.005 31707087 PMC7202961

[14] Yang Y, Klionsky DJ. Autophagy and disease: unanswered questions. Cell Death Differ. 2020; 27:858–71. 10.1038/s41418-019-0480-9 31900427 PMC7206137

[15] Mowers EE, Sharifi MN, Macleod KF. Autophagy in cancer metastasis. Oncogene. 2017; 36:1619–30. 10.1038/onc.2016.333 27593926 PMC5337449

[16] Towers CG, Wodetzki D, Thorburn A. Autophagy and cancer: Modulation of cell death pathways and cancer cell adaptations. J Cell Biol. 2020; 219:e201909033. 10.1083/jcb.201909033 31753861 PMC7039213

[17] Lambrechts D, Wauters E, Boeckx B, Aibar S, Nittner D, Burton O, Bassez A, Decaluwé H, Pircher A, Van den Eynde K, Weynand B, Verbeken E, De Leyn P, et al. Phenotype molding of stromal cells in the lung tumor microenvironment. Nat Med. 2018; 24:1277–89. 10.1038/s41591-018-0096-5 29988129

[18] Singer S, Malz M, Herpel E, Warth A, Bissinger M, Keith M, Muley T, Meister M, Hoffmann H, Penzel R, Gdynia G, Ehemann V, Schnabel PA, et al. Coordinated expression of stathmin family members by far upstream sequence element-binding protein-1 increases motility in non-small cell lung cancer. Cancer Res. 2009; 69:2234–43. 10.1158/0008-5472.CAN-08-3338 19258502

[19] Kaizuka T, Morishita H, Hama Y, Tsukamoto S, Matsui T, Toyota Y, Kodama A, Ishihara T, Mizushima T, Mizushima N. An Autophagic Flux Probe that Releases an Internal Control. Mol Cell. 2016; 64:835–49. 10.1016/j.molcel.2016.09.037 27818143

[20] Klionsky DJ, Abdelmohsen K, Abe A, Abedin MJ, Abeliovich H, Acevedo Arozena A, Adachi H, Adams CM, Adams PD, Adeli K, Adhihetty PJ, Adler SG, Agam G, et al. Guidelines for the use and interpretation of assays for monitoring autophagy (3rd edition). Autophagy. 2016; 12:1–222. 10.1080/15548627.2015.1100356 26799652 PMC4835977

[21] Sarkar S, Davies JE, Huang Z, Tunnacliffe A, Rubinsztein DC. Trehalose, a novel mTOR-independent autophagy enhancer, accelerates the clearance of mutant huntingtin and alpha-synuclein. J Biol Chem. 2007; 282:5641–52. 10.1074/jbc.M609532200 17182613

[22] Wu H, Huang S, Chen Z, Liu W, Zhou X, Zhang D. Hypoxia-induced autophagy contributes to the invasion of salivary adenoid cystic carcinoma through the HIF-1α/BNIP3 signaling pathway. Mol Med Rep. 2015; 12:6467–74. 10.3892/mmr.2015.4255 26323347 PMC4626194

[23] Amaravadi RK, Yu D, Lum JJ, Bui T, Christophorou MA, Evan GI, Thomas-Tikhonenko A, Thompson CB. Autophagy inhibition enhances therapy-induced apoptosis in a Myc-induced model of lymphoma. J Clin Invest. 2007; 117:326–36. 10.1172/JCI28833 17235397 PMC1765515

[24] Pankiv S, Clausen TH, Lamark T, Brech A, Bruun JA, Outzen H, Øvervatn A, Bjørkøy G, Johansen T. p62/SQSTM1 binds directly to Atg8/LC3 to facilitate degradation of ubiquitinated protein aggregates by autophagy. J Biol Chem. 2007; 282:24131–45. 10.1074/jbc.M702824200 17580304

[25] Hoang LT, Domingo-Sabugo C, Starren ES, Willis-Owen SAG, Morris-Rosendahl DJ, Nicholson AG, Cookson WOCM, Moffatt MF. Metabolomic, transcriptomic and genetic integrative analysis reveals important roles of adenosine diphosphate in haemostasis and platelet activation in non-small-cell lung cancer. Mol Oncol. 2019; 13:2406–21. 10.1002/1878-0261.12568 31461552 PMC6822241

[26] Zhang X, Nguyen KD, Rudnick PA, Roper N, Kawaler E, Maity TK, Awasthi S, Gao S, Biswas R, Venugopalan A, Cultraro CM, Fenyö D, Guha U. Quantitative Mass Spectrometry to Interrogate Proteomic Heterogeneity in Metastatic Lung Adenocarcinoma and Validate a Novel Somatic Mutation CDK12-G879V. Mol Cell Proteomics. 2019; 18:622–41. 10.1074/mcp.RA118.001266 30617155 PMC6442362

[27] Poillet-Perez L, White E. Role of tumor and host autophagy in cancer metabolism. Genes Dev. 2019; 33:610–19. 10.1101/gad.325514.119 31160394 PMC6546058

[28] Singh SS, Vats S, Chia AY, Tan TZ, Deng S, Ong MS, Arfuso F, Yap CT, Goh BC, Sethi G, Huang RY, Shen HM, Manjithaya R, Kumar AP. Dual role of autophagy in hallmarks of cancer. Oncogene. 2018; 37:1142–58. 10.1038/s41388-017-0046-6 29255248

[29] Koppenol WH, Bounds PL, Dang CV. Otto Warburg's contributions to current concepts of cancer metabolism. Nat Rev Cancer. 2011; 11:325–37. 10.1038/nrc3038 21508971

[30] Guo JY, Karsli-Uzunbas G, Mathew R, Aisner SC, Kamphorst JJ, Strohecker AM, Chen G, Price S, Lu W, Teng X, Snyder E, Santanam U, Dipaola RS, et al. Autophagy suppresses progression of K-ras-induced lung tumors to oncocytomas and maintains lipid homeostasis. Genes Dev. 2013; 27:1447–61. 10.1101/gad.219642.113 23824538 PMC3713426

[31] Rao S, Tortola L, Perlot T, Wirnsberger G, Novatchkova M, Nitsch R, Sykacek P, Frank L, Schramek D, Komnenovic V, Sigl V, Aumayr K, Schmauss G, et al. A dual role for autophagy in a murine model of lung cancer. Nat Commun. 2014; 5:3056. 10.1038/ncomms4056 24445999

[32] Kalluri R, Zeisberg M. Fibroblasts in cancer. Nat Rev Cancer. 2006; 6:392–401. 10.1038/nrc1877 16572188

[33] Viallard C, Larrivée B. Tumor angiogenesis and vascular normalization: alternative therapeutic targets. Angiogenesis. 2017; 20:409–26. 10.1007/s10456-017-9562-9 28660302

[34] Park JS, Burckhardt CJ, Lazcano R, Solis LM, Isogai T, Li L, Chen CS, Gao B, Minna JD, Bachoo R, DeBerardinis RJ, Danuser G. Mechanical regulation of glycolysis via cytoskeleton architecture. Nature. 2020; 578:621–26. 10.1038/s41586-020-1998-1 32051585 PMC7210009

[35] Totaro A, Zhuang Q, Panciera T, Battilana G, Azzolin L, Brumana G, Gandin A, Brusatin G, Cordenonsi M, Piccolo S. Cell phenotypic plasticity requires autophagic flux driven by YAP/TAZ mechanotransduction. Proc Natl Acad Sci U S A. 2019; 116:17848–57. 10.1073/pnas.1908228116 31416916 PMC6731754

[36] Bakir B, Chiarella AM, Pitarresi JR, Rustgi AK. EMT, MET, Plasticity, and Tumor Metastasis. Trends Cell Biol. 2020; 30:764–76. 10.1016/j.tcb.2020.07.003 32800658 PMC7647095

[37] Hamurcu Z, Delibaşı N, Geçene S, Şener EF, Dönmez-Altuntaş H, Özkul Y, Canatan H, Ozpolat B. Targeting LC3 and Beclin-1 autophagy genes suppresses proliferation, survival, migration and invasion by inhibition of Cyclin-D1 and uPAR/Integrin β1/Src signaling in triple negative breast cancer cells. J Cancer Res Clin Oncol. 2018; 144:415–30. 10.1007/s00432-017-2557-5 29288363 PMC11813384

[38] Tong H, Yin H, Hossain MA, Wang Y, Wu F, Dong X, Gao S, Zhan K, He W. Starvation-induced autophagy promotes the invasion and migration of human bladder cancer cells via TGF-β1/Smad3-mediated epithelial-mesenchymal transition activation. J Cell Biochem. 2019; 120:5118–27. 10.1002/jcb.27788 30320898

[39] Zhang L, Liu X, Song L, Zhai H, Chang C. MAP7 promotes migration and invasion and progression of human cervical cancer through modulating the autophagy. Cancer Cell Int. 2020; 20:17. 10.1186/s12935-020-1095-4 31956295 PMC6958635

[40] Catalano M, D'Alessandro G, Lepore F, Corazzari M, Caldarola S, Valacca C, Faienza F, Esposito V, Limatola C, Cecconi F, Di Bartolomeo S. Autophagy induction impairs migration and invasion by reversing EMT in glioblastoma cells. Mol Oncol. 2015; 9:1612–25. 10.1016/j.molonc.2015.04.016 26022108 PMC5528793

[41] Qiang L, Zhao B, Ming M, Wang N, He TC, Hwang S, Thorburn A, He YY. Regulation of cell proliferation and migration by p62 through stabilization of Twist1. Proc Natl Acad Sci U S A. 2014; 111:9241–46. 10.1073/pnas.1322913111 24927592 PMC4078859

[42] Wang Y, Xiong H, Liu D, Hill C, Ertay A, Li J, Zou Y, Miller P, White E, Downward J, Goldin RD, Yuan X, Lu X. Autophagy inhibition specifically promotes epithelial-mesenchymal transition and invasion in RAS-mutated cancer cells. Autophagy. 2019; 15:886–99. 10.1080/15548627.2019.1569912 30782064 PMC6517269

[43] Lambert AW, Pattabiraman DR, Weinberg RA. Emerging Biological Principles of Metastasis. Cell. 2017; 168:670–91. 10.1016/j.cell.2016.11.037 28187288 PMC5308465

