# Supplementary material for: Autophagic flux in cancer cells at the invasive front in the tumor-stroma border
Source: Aging (Albany NY). 2021 Aug 17;13(16):20229–45. doi: 10.18632/aging.203406 (PMC8436923; doi:10.18632/aging.203406)
Supplement: Supplementary Figures [file aging-13-203406-s001.pdf]

SUPPLEMENTARY FIGURES

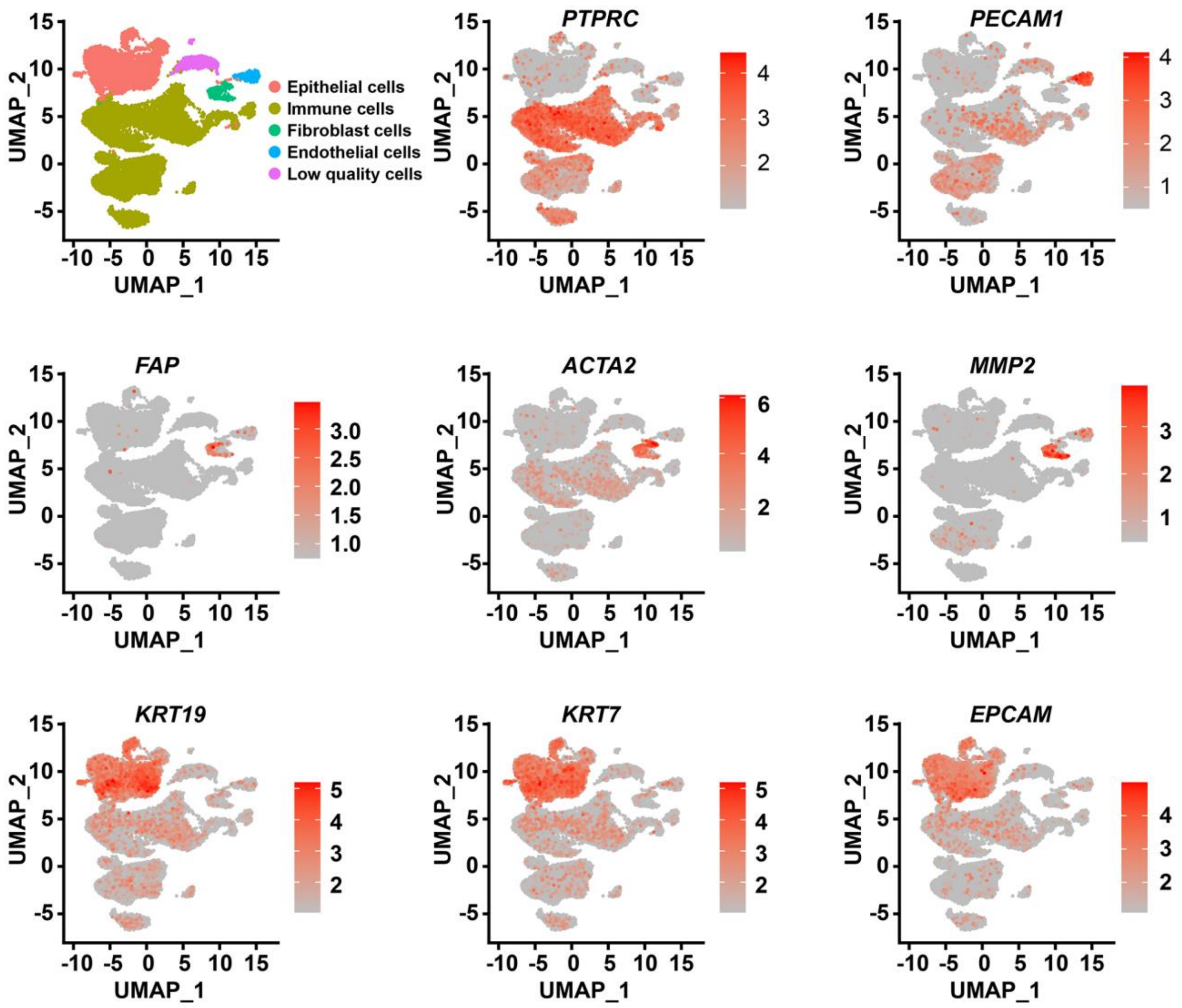

Supplementary Figure 1. Transcriptionally distinct cell types were identified by the transcripts of canonical cell type marker genes (Immune cells: *PTPRC*, endothelial cells: *PECAM1*, fibroblast cells: *FAP*, *ACTA2*, *MMP2*, epithelial cells: *KRT19*, *KRT7*, *EPCAM*).

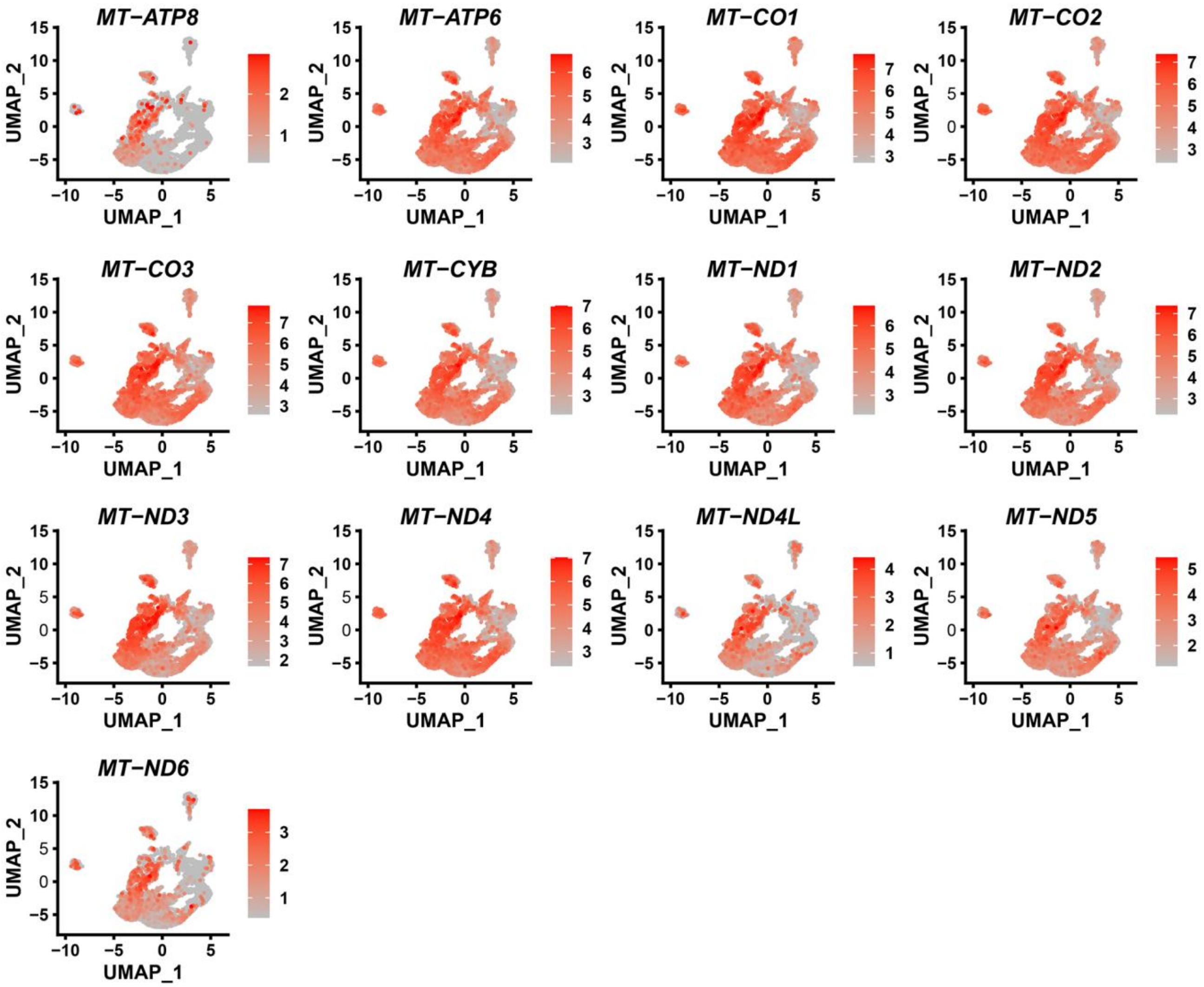

Supplementary Figure 2. UMAP visualization of MT-gene signature scores of malignant cells in 6 primary LUADs.
